# Modulating the Curvature of Protein Self-Assembled Spiral Nanotubules

**DOI:** 10.1021/acsami.5c01405

**Published:** 2025-05-12

**Authors:** Ariel Cohen, Itai Ben-Nun, Raviv Dharan, Tamar Tayri-Wilk, Asaf Shemesh, Avi Ginsburg, Abigail Millgram, Yael Levi-Kalisman, Israel Ringel, Uri Raviv

**Affiliations:** † Institute of Chemistry, 26742 The Hebrew University of Jerusalem, Edmond J. Safra Campus, Givat Ram, Jerusalem 9190401, Israel; ‡ The Harvey M. Krueger Family Center for Nanoscience and Nanotechnology, The Hebrew University of Jerusalem, Edmond J. Safra Campus, Givat Ram, Jerusalem 9190401, Israel; § Institute for Drug Research, The School of Pharmacy, Faculty of Medicine, The Hebrew University of Jerusalem, Ein Karem, Jerusalem 9112102, Israel

**Keywords:** SAXS, colchicine, spermine, tubulin, microtubule, cryo-TEM, conical spirals, helical structures

## Abstract

Structural transformations from ribbons to twisted ribbons to helical ribbons are often observed across supramolecular assemblies and macroscopic structures and can be described under a consistent theoretical framework. Conical molecular self-assembled structures, however, are rarely observed, may require more than one subunit, their dimensions are hard to control, and are poorly understood. Cytoskeleton microtubule (MT) is a dynamic protein–polymer that self-assembles from αβ-tubulin heterodimer, providing mechanical support to Eukaryotic cells. Colchicine is a drug known to bind the exchangeable nucleotide site on the β-tubulin subunit and suppress MT assembly. The tetravalent polyamine spermine promotes MT assembly and tubulin spiral structures, including conical tubulin spirals, tubules of conical spirals, and inverted helical tubules. Here we show how colchicine as a single agent suppressed MT and tubulin single ring assembly already at substoichiometric concentrations, whereas in the presence of spermine, the tubulin-colchicine stoichiometry controlled the dimensions and curvature of tubulin spiral assemblies. At a fixed spermine concentration, the concentration of colchicine modulated the radii of the nanotubular structures. The radii of the inverted helical nanotubules and conical spiral nanotubules monotonically decreased with colchicine concentration. We attribute our observation to the increased curvature of the tubulin dimer subunit induced by colchicine.

## Graphical abstract

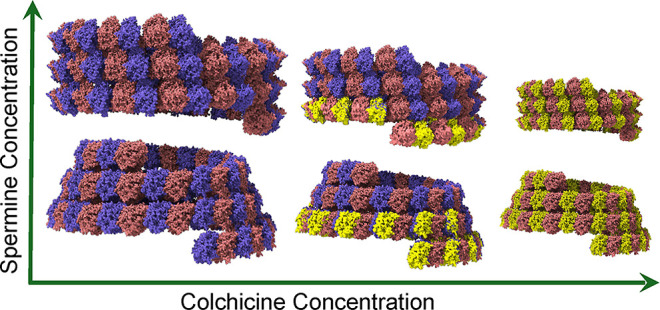

## Introduction

Revealing the architecture of macromolecular structures is of utmost importance for understanding their mechanism of action. Under the proper experimental conditions, a molecular structure may serve as a subunit for spontaneous and reversible association into organized structures in a process defined as self-assembly. However, correlating molecular properties and supramolecular morphology is currently limited. Macromolecular structures such as ribbons, twisted ribbons, and helical ribbons were observed as the product of the self-assembly of lipids, peptides, or proteins.^1,2^ Recent advances in polymer chemistry enabled the manipulation of helical handedness, pitch, and persistence length through chiral catalysts and controlled polymerization conditions.^3–6^ However, the precise control of the helical parameters in self-assembled structures remained a significant challenge in synthetic and biological systems.^7,8^

Self-assembled helical conical spiral structures were recently observed when the negatively charged αβ-tubulin heterodimer protein was incubated with the tetravalent polyamine spermine.^9^ The conformation and spontaneous curvature of the tubulin subunits determined the dimensions of the spiral structures. Yet, controlling the curvature of the helical and conical spiral tubular structures remained an open challenge.

Both in vivo and in vitro, tubulin can self-assemble into microtubule (MT), playing a key role in cell division, signaling, organelle transport, cell motility, and nerve and axonal function and stability.^10^ Tubulin is, therefore, an important and one of the best-validated targets for several clinically used drugs, including colchicine for gout, and the anticancer drugs paclitaxel (taxol), vincristine, and vinblastine.^11–14^

Strong head-to-tail (longitudinal) interactions between tubulin dimers form protofilaments, and weaker side-to-side (lateral) interactions hold protofilaments together, creating nanotubules, ∼25 nm in diameter.^15,16^ The activity, stability, and dynamics of MT mostly depend on whether guanosine triphosphate (GTP) or guanosine diphosphate (GDP) is associated at the exchangeable (E) nucleotide site located at β-tubulin monomer and facilitated by the GTPase activity of tubulin. The structure of tubulin was solved a long time ago.^17^ However, recent structural studies provide deeper insight into specific conformational changes in tubulin dimers, associated with GTP hydrolysis and dynamic instability.^16,18–28^ GDP-tubulin assembles into inverted tubulin single rings but does not polymerize into MTs.^26,28^ In polymerization buffer, GTP-tubulin above a critical concentration and temperature forms MT.^29^ At low temperatures, both GDP and GTP-tubulin assemble into linear (1D) curved oligomers and single tubulin rings.^26,28–31^ The standard self-association free energy and the mass fraction of tubulin in each assembly vary with nucleotide composition^26^ and so does the assembly rate of tubulin into single tubulin rings.^26,32,33^

Polyamines alter the polymerization of tubulin both in vitro and in cells in several ways.^34^ At low concentrations, polyamines stabilize the MT structure and dramatically increase the mass fraction of dimers that form the polymer.^34–36^ At higher spermine concentrations, GDP-tubulin forms in vitro an array of structures, depending on the polyamine structure and concentration.^9,36–38^ Drugs like colchicine or taxol may influence the assembly process as they can change the tubulin dimer structure and the association between dimers.^18,38–40^

Tubulin has two colchicine binding sites: a high-affinity site (*K*_D_ < 5 μM, generally accepted as the site of colchicine action) and a low-affinity site (*K*_D_ ∼ 650 μM) with which free colchicine rapidly exchanges (>100 s^–1^).^41,42^ When colchicine complexes with tubulin dimers at the high-affinity site, it blocks the GTP at the E-site on the β-tubulin and changes the curvature of the dimer.^43^ As both the tubulin curvature and the GTP at the E-site are crucial for MT assembly, the MTs disassemble without the ability to assemble again. Therefore, colchicine is an effective drug at low concentrations (1.2–2.4 mg/day) but becomes toxic at high concentrations (>0.5 mg/kg).^44^

In this paper, we incubated tubulin and colchicine at increasing colchicine/tubulin stoichiometry and formed an irreversible tubulin–colchicine complex. We examined the effect of the complex on tubulin assembly in the absence and presence of spermine. We discovered how increasing the colchicine concentration, at a fixed tubulin concentration, suppressed the assembly of tubulin rings and MTs and modulated the curvature of the spermine-induced helical and conical spiral nanotubules.^9,37,38^ So far, conical structures have not been predicated by current elastic theories.^2,45–47^ Modulating experimentally the dimensions of these structures provides a means to develop and examine advanced elastic theories of self-assembled structures that predict conical spiral structures.

## Results and Discussion

### Effect of Colchicine on Tubulin Assembly

We initially examined how colchicine modulates the steady-state assembly of GTP-tubulin, below (9 °C) and above (36 °C) the MT assembly temperature (Figures 1 and 2).

**Figure 1:**
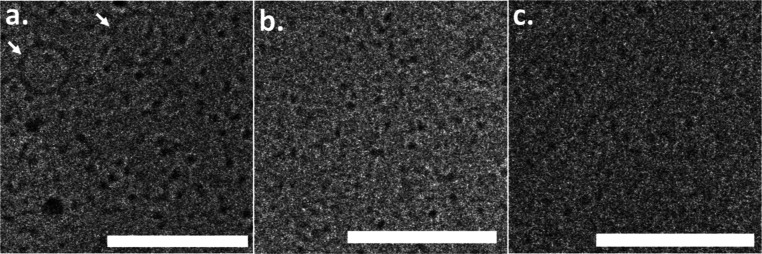
Effect of colchicine on tubulin single ring assembly. Selected cryo-TEM images of 100 μM GTP-tubulin incubated with 4 mM GTP for 2 h at 9 °C in the absence of colchicine (a), with 50 μM tubulin and 50 μM tubulin–colchicine complex (b), or with 100 μM tubulin–colchicine complex (c). The arrows point to tubulin single rings. Scale bars equal 100 nm. Figure S1 presents additional images. Two independent samples were measured in each case.

**Figure 2:**
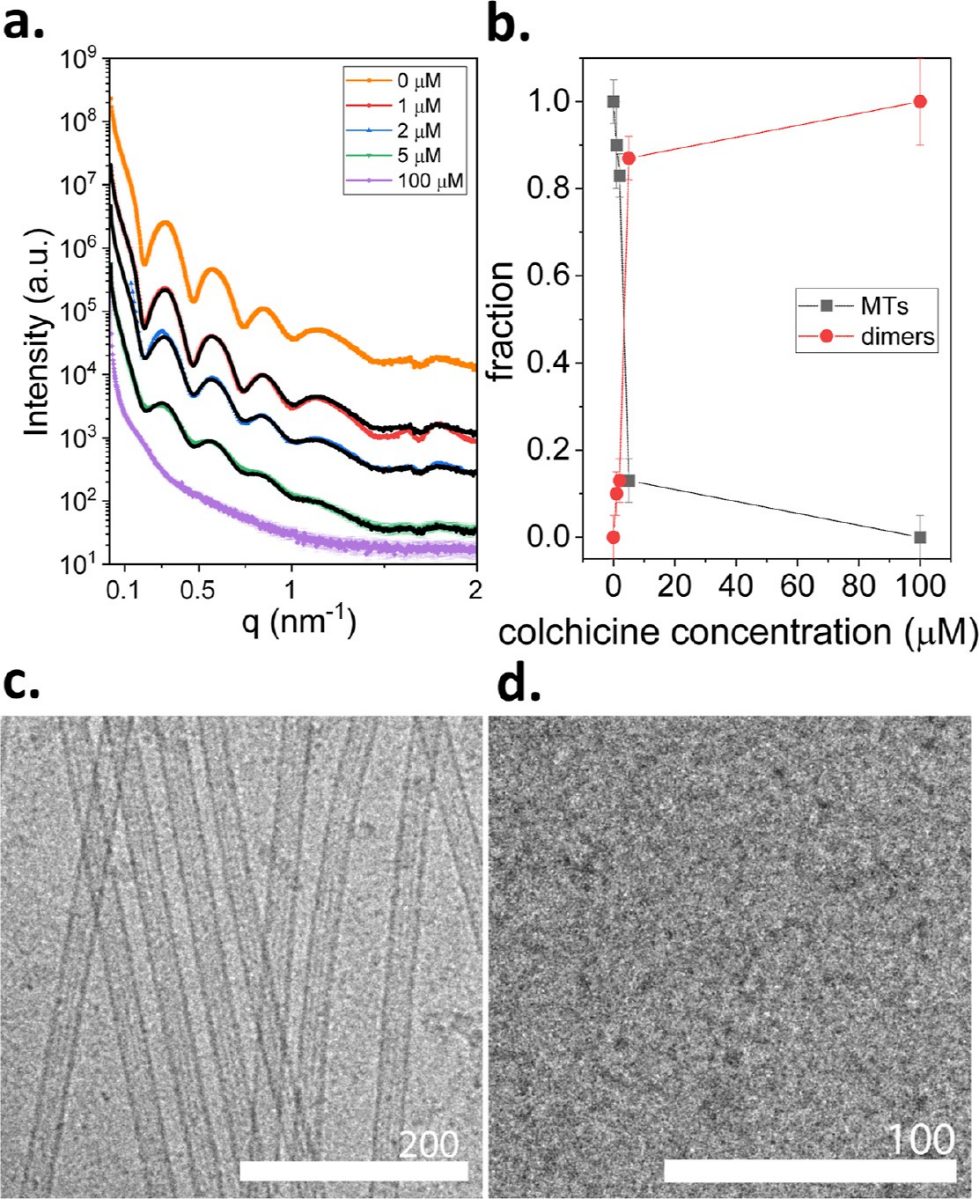
Effect of colchicine on MT assembly. (a) Azimuthally integrated supernatant-subtracted solution Small-Angle-X-ray Scattering (SAXS) intensity, *I*, as a function of the magnitude of the scattering vector, *q*, from 100 μM GTP-tubulin at different colchicine concentrations after ∼30 min incubation at 36 °C. We performed the measurements as explained in subsection SAXS Measurements in Materials and Methods. The scattering data were fitted to linear combinations (black curves) of the measured SAXS data when no colchicine was added (orange curve) and when 100 μM colchicine was added (purple curve). The colors of the error bars were matched to the color of the data and are shown in transparent mode. (b) The fractions of the MT-rich, no-added colchicine state (orange curve in a) and the tubulin-rich state (purple curve in a) as a function of colchicine concentration. (c) Cryo-TEM image of 100 μM GTP-tubulin solution at 36 °C (d) Cryo-TEM image of a solution of 100 μM GTP-tubulin and 100 μM colchicine at 36 °C. The number of independent measurements was 2 in (a) and 3 in (c and d).

At low temperatures, in the absence of colchicine, GTP-tubulin single rings coexisted with tubulin dimers and ring fragments (Figure 1a). After tubulin was incubated with colchicine and formed a tubulin–colchicine complex, tubulin single rings were not observed (Figure 1b,c). In the absence of colchicine at 36 °C, MT assembled, as expected (Figure 2a, orange curve, and Figure 2c). When we increased the colchicine/tubulin molar ratio, the typical MT form-factor had a smaller contribution to the scattering curves (Figure 2a). Accordingly, the fraction of the MT-rich phase monotonically decreased (Figure 2b), as expected.^48^ Colchicine effectively blocked tubulin assembly as it binds to the GTP at the N-site of β-tubulin,^49,50^ which is crucial for longitudinal dimer–dimer association, needed for tubulin single ring and MT assembly. At 1:1 tubulin/colchicine stoichiometry (i.e., at 100 μM colchicine), colchicine blocked the longitudinal association of tubulin dimers and neither tubulin single rings (Figure 1) nor MTs assembled (Figure 2). The concentration of tubulin oligomers was also negligible. As shown later, colchicine also blocked the longitudinal assembly of GDP-tubulin.

The analysis suggests that only a small fraction of MTs were assembled at substoichiometric colchicine concentrations (Figure 2a,b). In addition, the assembled MT formed with a distribution of protofilament numbers that was shifted to a larger fraction with 13 protofilaments (Figure S4 and Table S1).

This result is consistent with the binding of colchicine at a location inducing a curved conformation, thereby preventing the straight tubulin conformation needed for MT assembly.^40^ The shift to a smaller number of protofilaments suggests that colchicine slightly changed the tubulin curvature in the equatorial direction in addition to the longitudinal direction.

We investigated the assembly kinetics of 100 μM GTP-tubulin following a temperature change from 5 to 36 °C in the absence of colchicine and the presence of 2 μM colchicine (Figure 3). This was done at the ID02 time-resolved SAXS beamline, equipped with a stopped-flow device.^51^

**Figure 3:**
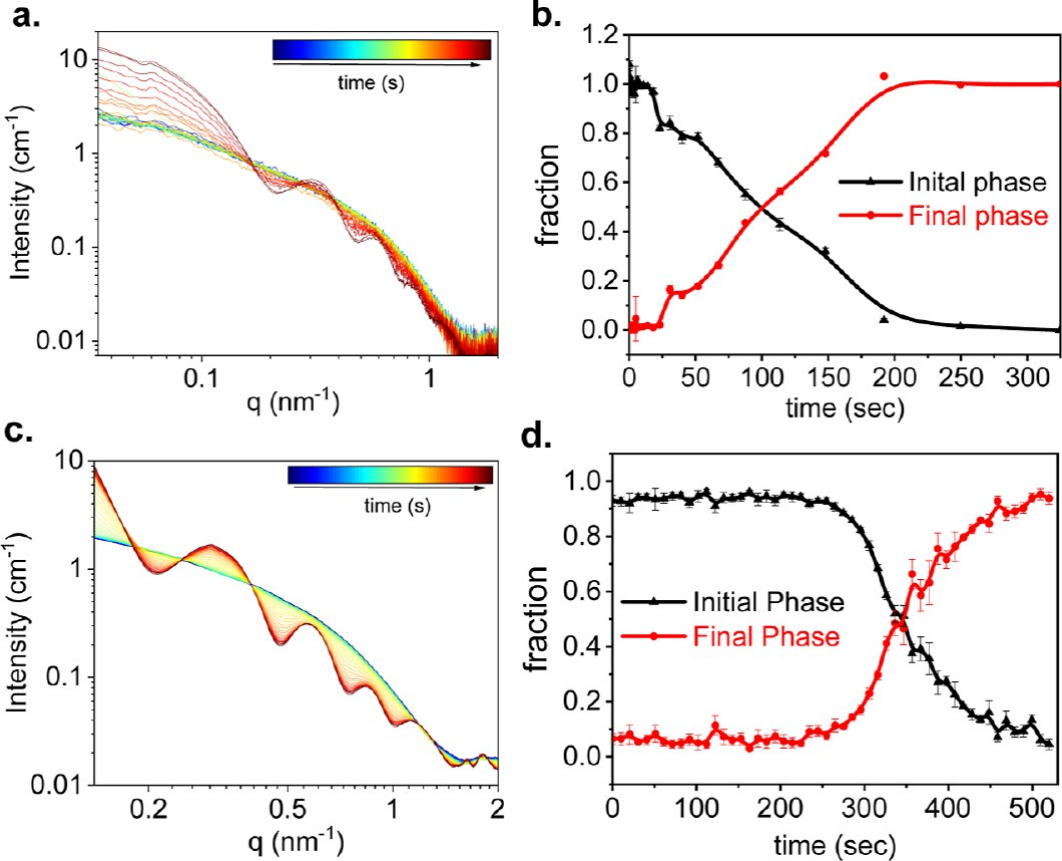
Time-resolved SAXS analysis of 100 μM GTP-tubulin assembly, induced by a temperature rise from 5 to 36 °C (a and b). The experiment was repeated in the presence of 2 μM colchicine (c and d). As demonstrated in Figure S2, the data were fitted to a linear combination of the initial tubulin-rich state and the final MT-rich state. The fraction of the two states is plotted as a function of the time elapsed after the temperature change (b and d). Curves with stronger oscillations correspond to states containing a larger fraction of MTs (approaching the final phase). Less oscillatory curves correspond to states containing a larger fraction of tubulin dimers (closer to the initial phase). The two-state model is also consistent with an SVD analysis (Figure S3). The number of independent measurements was 5 in (a) and 2 in (c). For the clarity of presentation, representative measurement error bars in (a and c) are shown in Figure S2.

Our earlier studies showed that in the absence of colchicine, cold GTP-tubulin solution includes coexisting dimers, 1D (linear) curved oligomers (or ring fragments), and tubulin single rings.^52,53^ When the temperature increased, these structures assembled into MTs (Figure 3a). When the assembly experiment was repeated in the presence of 2 μM colchicine, the scattering curves had isosbestic points at which the curves crossed each other (Figure 3c). Isosbestic points are typically observed when the scattering curves of the contributing species have the same intensity at specific scattering angles. Hence, during a process, when the fractions of species vary, the intensities are fixed at those specific scattering angles. Statistically, it is unlikely to have more than two species with the same scattering intensity at specific scattering angles. Our observation of isosbestic points, therefore, indicates a two-state reaction.^54,55^ The entire data set fitted well (*R* ^2^ between 0.98 and 0.99) to a linear combination of the tubulin dimer-rich initial scattering curve and the MT-rich final curve. Examples of the fitting results are shown in Figure S2. Singular value decomposition (SVD) analysis (Figure S3) indeed shows that two states are sufficient to reproduce the entire data set. From the fit (Figure S2), the fractions of the initial and the final states were determined (Figure 3d). The two-state mechanism was obtained at a remarkably low substoichiometric molar ratio of colchicine and tubulin (1:50).

The initial lag phase is consistent with our earlier two-state assembly mechanism of Hepatitis B virus capsid assembly, analyzed by time-resolved SAXS.^54,56^ The lag phase and two-state mechanism suggest a weak dimer–dimer association standard free energy and a high energy barrier for assembly, followed by a downhill energy landscape without deep local minima. Intermediates did not accumulate to a detectable amount (about 1% of the total tubulin mass), because they either passed the assembly barrier and elongated or completely disassembled.

In the absence of colchicine, isosbestic points were not observed, and the lag phase was shorter (Figure 3a,b). SVD analysis (Figure S3) shows that additional intermediates accumulated during the assembly reaction were required to analyze the data. These results are consistent with stronger dimer–dimer association standard free energies and lower energy barriers for assembly.^54,57^ A complete analysis of these data, however, is beyond the scope of this paper. Instead, we fitted the contribution of the initial and final states at each time point along the reaction (Figure 3b). The *R* ^2^ value, however, was only 0.87, consistent with the SVD analysis, indicating additional intermediates formed during the reaction.

The association of colchicine with the tubulin dimer blocked the GTP at the E-site of the β-tubulin and limited the longitudinal association between dimers.^40^ Hence, the assembly of the intermediates observed in the absence of colchicine, was significantly blocked already when 2 μM colchicine was added. SVD analysis suggests that at 1 μM colchicine, intermediates could still be detected (Figure S3).

### Effect of Colchicine on the Assembly of Tubulin in the Presence of Spermine

To further understand the effect of colchicine on tubulin assembly, we examined its effect on other types of structures formed by GDP-tubulin. GDP-tubulin can assemble into tubulin single rings^26^ at low temperatures but cannot assemble into MT at 36 °C, hence the competition with MT assembly was eliminated in those experiments. Recently,^9^ we have shown that a solution of GDP-tubulin and spermine can self-associate into a range of hierarchical structures. At low spermine concentrations (1–2.5 mM) GDP-tubulin assembles into conical spirals, containing between 1 and 3 helical turns. At 5 mM spermine, conical spirals with three helical turns assemble into conical spiral tubules, which bundle in a combination of antiparallel and parallel associations. Further increase of the spermine concentration to 10 mM leads to inverted helical tubules. In all of these structures, the tubulin side facing the MT lumen is facing the external side of the helical and conical structures.^9,37^

We complexed cold (9 °C) GDP-tubulin with colchicine at a 1:1 stoichiometric molar ratio (see subsection Colchicine–Tubulin Complex) and mixed it with spermine solutions at increasing spermine concentrations. In the absence of spermine, only tubulin dimers were observed as colchicine blocked the formation of the small tubulin assemblies, including tubulin single rings and curved linear (1D) tubulin oligomers (Figure 4a). In other words, colchicine blocked any longitudinal association between GDP-tubulin dimers.

**Figure 4:**
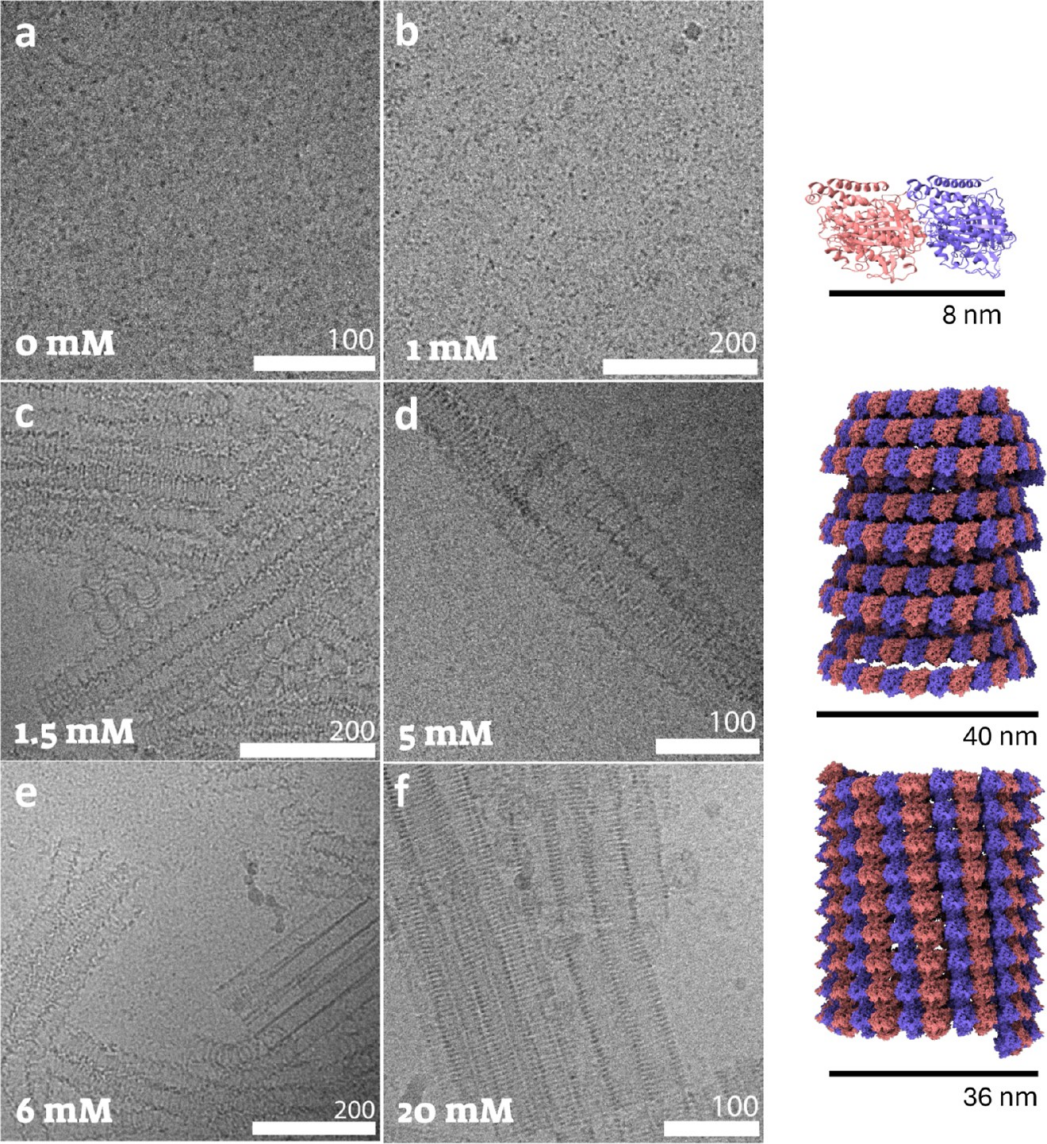
Cryo-TEM images of 40 μM GDP-tubulin–colchicine complex at a 1:1 molar ratio after 48 h incubation at 9 °C with the indicated spermine concentrations (a–f). Atomic models of the dominant structures, dimer (top), left-handed conical spiral tubule (middle), and left-handed inverted helical tubules (bottom), are presented on the right. The models are based on SAXS analysis (Figures 5 and 7). Two independent samples were measured in each case.

When 1 mM spermine was added, the assembly of short conical spirals, formed in the absence of colchicine,^9^ was also blocked (Figure 4b). Long conical spiral tubules, however, formed already at 1.5 mM spermine (Figure 4c), instead of 5 mM in the absence of colchicine.^9^ This result is analogous to the two-state assembly mechanism of MT in the presence of colchicine (Figure 3) because dimers either did not assemble or passed the barrier and formed conical left-handed spiral tubules. Larger bundles of conical spiral tubules formed at 5 mM spermine (Figure 4d). Inverted left-handed helical tubules started to form already at 6 mM spermine (Figure 4e) and coexisted with conical spiral tubules, instead of 7.5 mM spermine in the absence of colchicine.^9^ At 20 mM spermine, only inverted helical tubules were observed (Figure 4f) as in the absence of colchicine. Note that at 36 °C, in the absence of colchicine, it was easier to pass the kinetic barriers and the formation of conical spiral tubules started at a lower spermine concentration (2.5 mM rather than 5 mM at 9 °C) and so does the formation of inverted tubules (which started already at 5 mM).^9^

The results show that in the absence of spermine, colchicine blocked longitudinal tubulin association not only at high temperatures (where MT assembles) but also at low temperatures, where tubulin assembles into single rings and ring fragments. However, when spermine was added, the tubulin-colchicine complex was able to associate longitudinally but only above a higher (1.5 mM) spermine concentration, and then only larger assemblies were detected (conical spiral tubules or inverted helical tubules but not short conical spirals). These observations suggest that colchicine prevented competition with MT or tubulin ring assemblies and effectively decreased the energy barriers for forming conical spiral tubules and inverted helical tubules.

Based on the results shown in Figure 4, we examined the effect of varying colchicine concentrations on the assembly of cold GDP-tubulin with either 5 mM spermine (Figure 4d), where mainly long conical spiral tubules formed or 20 mM spermine, where mainly inverted helical tubules formed (Figure 4f). In this way, we minimize the effects of heterogeneous bundle populations that would otherwise contribute overlapping signals to the scattering data.^9^ As tubulin has two colchicine binding sites, we gradually increased the colchicine: tubulin molar ratio from 0 to 2.^41^

The most notable effect of colchicine concentration was on the radii (or local curvature) of the conical spiral (Figures 5 and 6) and inverted helical tubules (Figure 7). Figure 8 shows the structural parameters of the conical spiral tubules. Figure S6 demonstrates how different structural parameters affect the SAXS model of conical spiral tubules. The most distinguishable structural parameters are the minimal and maximal radii (*R*_min_ and *R*_max_, respectively). By fitting the data to the models, we found that increasing the colchicine–tubulin stoichiometry decreased the radii of the conical spiral tubules (Figure 5b). This result is consistent with the curved dimer conformation induced by colchicine.^40,58^ Increasing the fraction of the more curved tubulin-colchicine complex, decreased the average radii of the conical spirals. The minimal and maximal radii are separated by vertical height, *V*_h_ (Table S2). The angle of the conical structure (defined in Figure 8), given by

1 $$\theta =\arctan\left(\frac{R_{\max}-R_{\min}}{V_{h}}\right)$$

slightly increased, from 21 in the absence of colchicine to 24° when the colchicine-tubulin molar ratio was 2 (Table S2).

**Figure 5:**
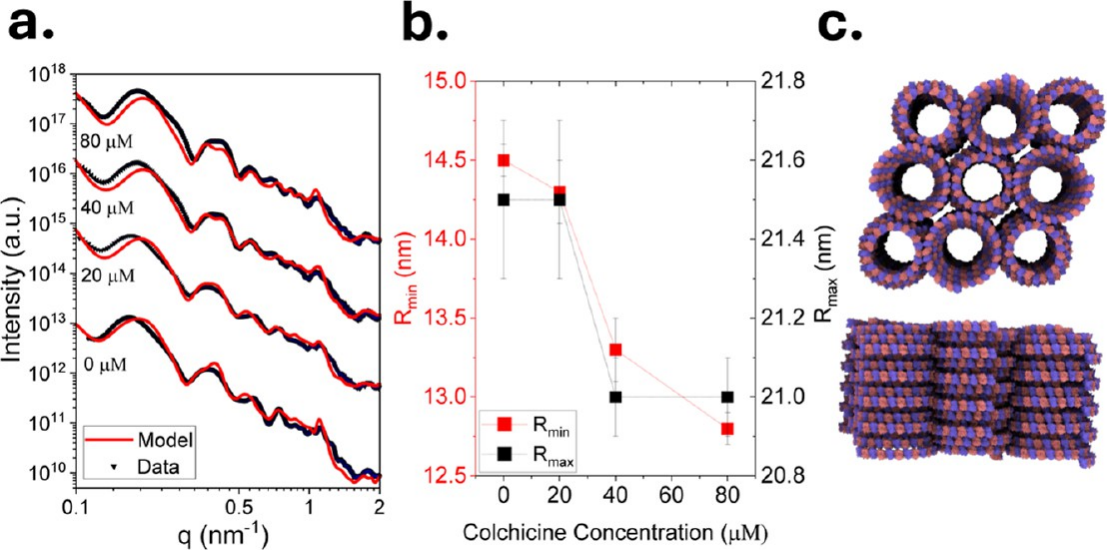
Modulating the curvature of conical spiral tubules using colchicine. 40 μM GDP-tubulin incubated with 5 mM spermine for 48 h at 9 °C with the indicated increasing colchicine concentration. (a) Supernatant-subtracted SAXS curves at different colchicine concentrations (black symbols and blue error bars), fitted to atomic models of bundles of left-handed conical spiral tubules (red curves). Two independent samples were measured in each case. (b) The minimal and maximal radii of the conical spirals of the bundles (analyzed in panel a) as a function of colchicine concentration. (c) Cartoons of the atomic model of the bundles of conical spiral tubules used to fit the data in a. The modeled bundles contained 3 × 3 or 4 × 4 conical spiral tubules, where the central tubule was surrounded by four antiparallel tubules and two parallel tubules. The structural parameters, illustrated in Figure 8, at each colchicine concentration are provided in Tables S4.

**Figure 6:**
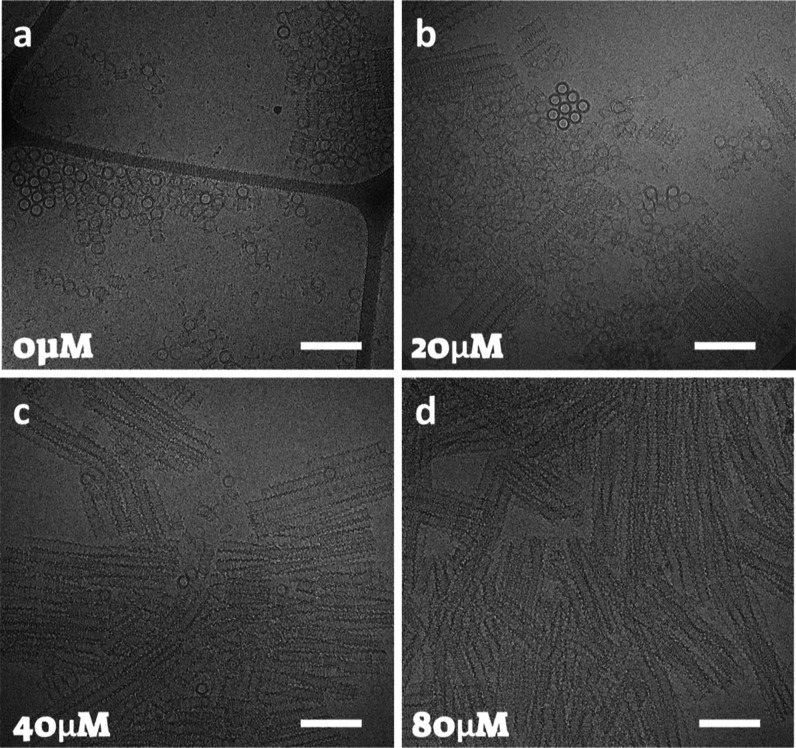
Cryo-TEM images of 40 μM GDP-tubulin incubated with 5 mM spermine for 48 h at 9 °C with the indicated colchicine concentration (a–d). Scale bars equal 200 nm. Three independent samples were measured in each case.

**Figure 7:**
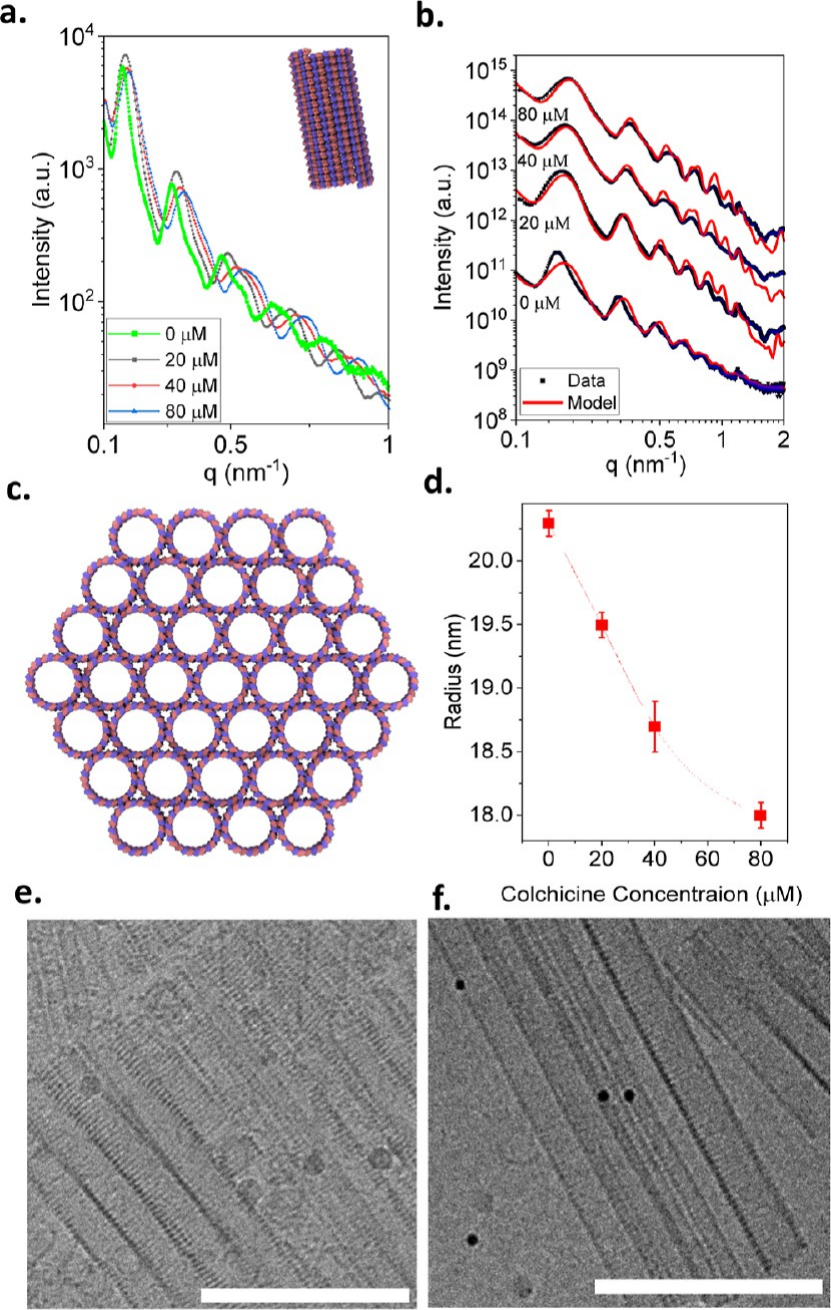
Modulating the curvature of inverted helical tubules using colchicine. (a) Supernatant-subtracted SAXS curves from 40 μM GDP-tubulin complexed with the indicated colchicine concentrations following 48 h incubation with 20 mM spermine at 9 °C. (b) The SAXS curves from a. (black symbols and blue error bars) were fitted to models (red curves), each comprising a linear combination of a free dimer, a short (10 nm) left-handed single inverted helical tubule, and a hexagonal bundle of long (100 nm) left-handed inverted helical tubules (illustrated in c) with bundle parameters of *n*_1_ = *n*_2_ = 4 (see Bundles and Inverted Helical Tubule Model). The mass fractions the contributing structures are presented in Table S6d. (d) The variation of the helical radii as a function of colchicine concentration. All the other structural parameters are shown in Tables S5 and S7. Selected cryo-TEM images of the structures formed with 40 μM colchicine (e) and in the absence of colchicine (f). Scale bars equal 200 nm. Three independent samples were measured in each case.

**Figure 8:**
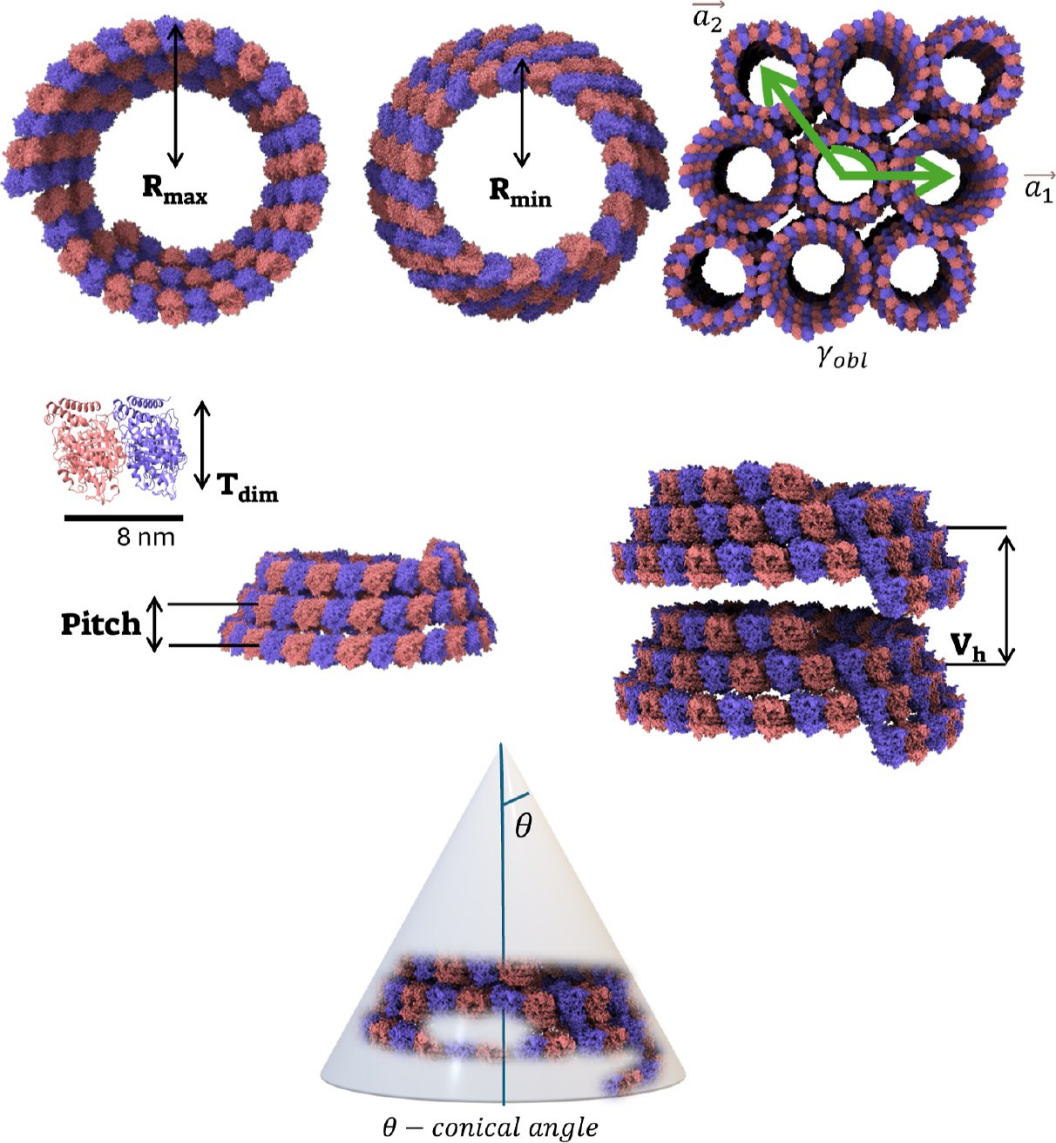
Cartoon of the conical spiral tubule structural parameters. *R*_max_ and *R*_min_ are the maximal and minimal radii of the conical spiral, θ is the conical angle formed by the spiral, the pitch, *p*, is the vertical rise per helical turn, and *V*_h_ is the repeat distance between the centers of successive conical spirals in the conical spiral tubule. ${\mathrm{a⃗}}_{1}$ and ${\mathrm{a⃗}}_{2}$ are the bundle lattice vectors. γ_obl_ is the angle between lattice vectors. The thickness of a tubulin dimer, *T*_dim_, and the height of a single conical spiral with 3 helical turns, *h*, were used as model constraints rather than structural model parameters.

We also found that by increasing the concentration of colchicine, the mass fraction of tubulin in short (15 nm) conical spiral tubules decreased, whereas the mass fraction of tubulin assembled into long (10 nm) conical spiral tubules increased (Figures 6 and S4). However, in inverted helical tubules (Figure 7), the mass fraction of short single inverted helical tubules considerably increased, and the mass fraction of large bundles decreased with colchicine concentration (Table S6).

In the presence of spermine, the mass fraction of free tubulin was low, owing to the much stronger dimer–dimer association standard free energy induced by spermine. This result significantly differs from the relatively high critical concentration of free tubulin dimers (∼20 μM) under conditions that lead to MT assembly.^29^ It means that spermine recruited most of the free tubulin into the tubular phases and significantly decreased the critical assembly concentration of tubulin.

As in the case of conical spirals, an increase in the local curvature between the α and β tubulin decreased the radius of the inverted helical tubules with elevated colchicine concentrations (Figure 7c).

Fitting to inverted helical tubules was based on testing how each structural parameter influences the scattering curves (Figure S7). A similar analysis was performed for conical spirals (Figure S6). In both cases, the scattering curves were very sensitive to the radii of the structures and the helical pitch.

In summary, by binding to the E-site of the nucleotide on the β-tubulin, colchicine blocks longitudinal tubulin association. Therefore, when spermine was added, the competition with the regular tubulin assemblies involving longitudinal dimer association (rings/MT) was eliminated. Effectively, the energy barriers for forming tubulin conical spiral tubules and inverted helical tubules were lower, as were the required minimal spermine concentrations. In addition, the tubules were longer than those obtained without colchicine, suggesting that elongation was more efficient than starting new lines of assemblies.

Our earlier study^9^ showed that putrescine dihydrochloride or spermidine trihydrochloride, containing only two or three amine groups, respectively, were insufficient to induce any of the tubular assemblies. Those assemblies were observed only with the tetraamines spermine or thermospermine.^9^

## Conclusions

In this paper, we examined the effect of colchicine as a modulator of tubulin assembly and, in the presence of spermine, as a modulator of the curvature of conical spiral and helical tubular polymeric architectures. In the absence of spermine, at 36 °C, colchicine blocked the assembly of microtubules and, at low temperatures, the assembly of tubulin single-ring and ring fragments. At 1%–2% colchicine/tubulin molar ratio, colchicine led to a two-state microtubule assembly mechanism with a long initial lag phase. This observation is consistent with no accumulation of intermediates because they either passed a high energy barrier and formed microtubules or completely disassembled. In the presence of spermine, we showed how increasing the concentration of colchicine decreased the radii of our conical spiral and helical assemblies. The fact that the small tubulin assemblies (rings and ring fragments) did not form or accumulate also eliminated the competition of spermine with these small assemblies and lowered the barrier to the formation of long conical spiral tubules or inverted tubulin tubules.

## Materials and Methods

### Sample Preparation

Tubulin was purified from bovine brains using our published protocol,^28^ and stored at −80 °C. Before experiments, tubulin was thawed slowly on ice for 30 min. The solution was centrifuged at 15700 *g* for 30 min at 4 °C. The top 85% of the supernatant was used for the experiments.^48^

### GDP-Tubulin

To get GDP-tubulin, we hydrolyzed the GTP to GDP by applying three 10 min polymerization/depolymerization cycles at 10 and 30 °C. Similar results were obtained when we applied three cycles at 4 and 30 °C, 30 min in each step.^9^ The tubulin was then centrifuged at 15700 *g* for 30 min at 4 °C. The top 85% of the supernatant was used for the experiments and diluted to 80 μM in PEM50 (50 mM PIPES, 1 mM EGTA, 1 mM MgCl_2_ and 5% v/v glycerol).

### GTP-Tubulin

To get GTP-Tubulin, an excess of 4 mM GTP was added directly to the purified tubulin, which did not undergo the polymerization/depolymerization cycles. The mixture was incubated for 30 min on ice or at 9 °C.

### Colchicine–Tubulin Complex

Ice cold tubulin was incubated at 36 °C for 30 min with colchicine at the required tubulin/colchicine stoichiometry.

### Tubulin-Spermine Assemblies

We prepared mixtures of GDP-tubulin with the corresponding tubulin-colchicine complex at different molar ratios. We mixed on ice equal volumes of 80 μM of the resulting tubulin solution with spermine tetrahydrochloride solution in PEM50 at twice the final spermine concentration. The solution was then incubated at 9 (Figures 5 and 7) or 36 °C (Figure S5) for different periods as indicated.

### SAXS Measurements

The SAXS measurements in Figures 2–4 were performed at ESRF (Grenoble) at ID02 (operated by T. Narayanan and his team).^51^ A volume of 30 μL was used for each steady-state measurement in a 2 mm thick flow-through quartz capillary cell. Between 10 and 20 frames were scanned, using an exposure time of 100 msec per scan. The scattering intensity was recorded on an Eiger2 4M detector. The sample-to-detector distance was 3 m, and the energy of the X-ray photons was 10 keV. Calibration to absolute intensity was done with water.^59^ In time-resolved kinetic measurements, a stopped-flow device was used (SFM4000/MS70, Biologic), and the buffer served as a background that was subtracted from the measurements.^51^ In steady-state measurements, we prepared at least 100 μL of each sample and used 30 μL to measure the sample. The remaining solution volume was centrifuged at 15700 *g* for 30 min at the measurement temperature, and the top 30 μL supernatant was measured and subtracted from the initial measurement of the sample. This measurement protocol was used and explained in our earlier studies.^60–62^ SAXS-Utilities was used to azimuthally integrate the 2D scattering data and subtract the background. The SAXS measurements presented in Figures 3, 5, and 7, developing the sample preparation protocols, and repeating the results were performed using our home-built SAXS setup,^63^ for which Silver behenate was used as a standard to determine the sample-to-detector distance.

At least two independent samples were prepared and measured in each case, as indicated in the relevant figure captions.

### Cryo-TEM

We imaged the samples using transmission electron microscopy at cryogenic temperatures (cryo-TEM). A 2.5 μL droplet was deposited on a 300-mesh copper lacey grid (Ted Pella Ltd.), equilibrated at 9 or 36 °C and 100% humidity inside a Vitrobot Mark IV. The sample was blotted by the vitrobot, creating ultrathin films with a thickness varying between 20 and 300 nm. Specimens were vitrified by rapid plunging into liquid ethane precooled with liquid nitrogen. The samples were then imaged, using a Tecnai G2 Spirit Twin T-12 TEM (Thermo-scientific), cooled by liquid nitrogen, and operated at an acceleration voltage of 120 keV in a low-dose mode. Images were recorded on a 4 K × 4 K FEI Eagle CCD camera with a defocus value between 2 and 4 μm.

At least two independent samples were prepared and measured in each case, as indicated in the relevant figure captions.

### Computed Models

The SAXS models are based on the central tubulin dimer atomic model, taken from the protein data bank (PDB) entry 3J6F as the subunit.^18^ When colchicine was added, the subunit was based on PDB4O2B.^58^ The dimers were centered, and hydrogen atoms were added by ChimeraX.^64^ The buffer electron density was 334 e/nm^3^ and the electron density of the hydration shell was 364 e/nm^3^ and its thickness was 0.28 nm. D+ software and its Python API^62,65^ were used to compute the scattering intensity, *I*, as a function of the magnitude of the momentum transfer, *q*. The Python code of all the models was deposited in a GitHub folder (https://github.com/Itaibnn/lab-projects/tree/main/Geometry%20structures). The Python code gets the radius, *r*, of the structure, the tubulin dimer width, *T*_dim_, and length, *L*_dim_, as constrained parameters, the pitch of the spiral structure, *p*, and the total height, *h*, of the tubular structure in nm units. In the case of MT, we also define the protofilament number, *N*_p_. In conical spiral tubules, we also included *R*_min_ and *R*_max_, the number of helical turns, the distance between subsequent conical repetitions, *V*_h_ in nm, and *N*_Spirals per tubule_ (Figure 8). To model the bundles, we define the length of the lattice vector, *a*, the angles between the lattice vectors, γ, and the number of repeating tubules, *n*_1_ and *n*_2_, in the two lattice directions (the exact parameter definitions are explained in the subsections below). The code computes the “dol” file providing the center of mass position (*x*,*y*,*z*) of each repeating dimer and its orientation (α,β,γ) using the Tait–Bryan rotation matrix convention^66^

2 $$A\left(\alpha ,\beta ,\gamma \right)=A_{x}\left(\alpha \right)A_{y}\left(\beta \right)A_{z}\left(\gamma \right)=\left[\begin{array}{lll} \cos\beta \cos\gamma & -\cos\beta \sin\gamma & \sin\beta \\ \cos\alpha \sin\gamma +\cos\gamma \sin\alpha \sin\beta & \cos\alpha \cos\gamma -\sin\alpha \sin\beta \sin\gamma & -\cos\beta \sin\alpha \\ \sin\alpha \sin\gamma -\cos\alpha \cos\gamma \sin\beta & \cos\gamma \sin\alpha +\cos\alpha \sin\beta \sin\gamma & \cos\alpha \cos\beta \end{array}\right]$$

where α, β, and γ, are the rotations about the *x*, *y*, and *z* axes, respectively. The scattering data and the cryo-TEM images guided our modeling.

### Inverted Helical Tubule Model

The inverted helical tubule model was based on a helical symmetry with left-handed chirality, described by

3 $$x_{i}=r\cos\left(\theta_{i}\right)$$

4 $$y_{i}=r\sin\left(\theta_{i}\right)$$

5 $$z_{i}=i\frac{pL_{\dim}}{L_{turn}}$$

6 $$L_{turn}=\sqrt{4\pi^{2}r^{2}+p^{2}}$$

7 $$\theta_{\mathrm{Dim}}=\frac{L_{\dim}}{r}$$

8 $$i_{\max}=[\frac{h}{p}\frac{L_{turn}}{L_{\dim}}+1],,i\in \left\{0,\;1,\;...,i_{\max}\right\}$$

9 $$\theta_{i}=i\theta_{\dim},\mathrm{mod}2\pi$$

$$\alpha_{i}=90^{{}^{\circ}}-\frac{180^{{}^{\circ}}}{\pi }\arcsin\frac{p}{L_{turn}}$$ 10

11 $$\beta_{i}=\frac{180^{{}^{\circ}}}{\pi }\theta_{i}$$

12 $$\gamma_{i}=0$$

where the parameters are defined as follows:

- *p*—The vertical pitch, defining the distance between the centers of successive helical turns.
- *h*—The total height of the helical structure.
- *L*_dim_—The length of a dimer.
- *L*_turn_—The length of a helical turn along the line connecting the centers of the dimers.
- *i*_max_—The total number of dimers in the helical tubule.
- θ_dim_—The azimuthal angle per dimer in the helical tubule (in radians).
- θ_*i*_—The total azimuthal angle of the *i*-th dimer in the helical tubule (in radians).
- α, β, and γ, are the Tait-Bryan rotation angles around the *x*, *y*, and *z* axes, respectively (in degrees).

### Conical Spiral Tubule Model

The conical spiral tubule model (Figure 8) was built based on a conical helix parametrization with left-handed chirality, described by

13 $$x_{i}=\left(R_{\max}-Ct_{i}\right)\cos t_{i}$$

14 $$y_{i}=\left(R_{\max}-Ct_{i}\right)\sin t_{i}$$

15 $$z_{i}=\frac{p}{2\pi }t_{i}$$

16 $$C=\frac{R_{\max}-R_{\min}}{2\pi n}$$

17 $$A=\frac{p}{2\pi }$$

18 $$S\left(t_{i}\right)=\frac{1}{2C}\left[\left(C^{2}+A^{2}\right)\mathrm{arcsinh}\left(\frac{C\left(Ct-R_{\max}\right)}{C\sqrt{C^{2}+A^{2}}}\right)+\left(Ct-R_{\max}\right)\sqrt{{\left(Ct-R_{\max}\right)}^{2}+C^{2}+A^{2}}\right]{|}_{0}^{t_{i}}$$

19 $$\alpha_{i}=90^{{}^{\circ}}-\frac{180^{{}^{\circ}}}{\pi }\arcsin\left(\frac{p}{2\pi L_{\dim}}\Delta t_{i}\right)$$

20 $$\beta_{i}=\frac{180^{{}^{\circ}}}{\pi }t_{i}$$

21 $$\gamma_{i}=0$$

The azimuthal angle (in radians), *t*_*i*_, at the center of the *i*-th tubulin dimer in the conical spiral tubule, was determined using a binary search within the range of 0 to 2π*n*, where *t*_0_ was set to 0. This search algorithm iteratively narrowed the interval around *t*_*i*_, satisfying the condition *L*_dim_ = *S*(*t*_*i*_) – *S*(*t*_*i*–1_) using a convergence tolerance of 10^–14^ nm. *S*(*t*_*i*_) is the total arc length from the start to the center of the *i*-th dimer along the conical spiral. A more detailed derivation is provided in section Derivation of the Conical Spiral Tubule Model in the Supporting Information.

We then duplicate the conical spiral basic unit along the *z*-axis with a vertical height, *V*_h_, between the centers of successive conical spirals. Finally, to ensure the center of mass of the tubules along the *z*-axis is at the origin, we shifted the *z*-axis by $-\frac{z_{\max}}{2}$ .

The other parameters are defined as follows:

- *p*—The vertical pitch of a single helical turn in a conical spiral.
- *V*_h_—the vertical distance between the centers of two consecutive conical spirals.
- *n*—The number of helical turns in a conical spiral.
- *R*_max_—The maximal radius to the center of mass of a dimer in a conical spiral (the largest radius).
- *R*_min_—The minimal radius to the center of mass of a dimer in a conical spiral (the smallest radius).
- *C*—The rate at which the radius decreases per radian along the conical spiral.
- Δ*t*_*i*_ ≡ *t*_*i*+1_ – *t*_*i*_. The azimuthal angle covered by the *i*-th dimer in the conical spiral structure.

### Bundles

The 2D hexagonal and oblique bundles (Figure 8) are defined by the repeating subunit vectors

22 $${\mathrm{R⃗}}_{n}=n_{1}{\mathrm{a⃗}}_{1}+n_{2}{\mathrm{a⃗}}_{2}$$

where

23 $${\mathrm{a⃗}}_{1}=a\left(1,0\right)$$

24 $${\mathrm{a⃗}}_{2}=a\left(\cos\gamma_{Lattice},\sin\gamma_{Lattice}\right)$$

and *a* is the center-to-center distance between neighboring helical tubules in the hexagonal lattice or neighboring antiparallel conical spiral tubules in the oblique lattice. In the oblique lattice of the conical spiral tubule bundle *a* ≥ *R*_max_ + *R*_min_ + *T*_dim_. *T*_dim_ is the dimer-thickness, ensuring the tubules did not penetrate each other. In the hexagonal bundle of the inverted helical tubules *a* ≥ 2*r* + *T*_dim_. In the hexagonal lattice γ_Hex_ = 120° and *n*_*i*_ varied between $-n_{i}^{\max}$ and $n_{i}^{\max}$ where *i* ∈ {1, 2}, as long as $∥{\mathrm{R⃗}}_{n}∥\leq a\left(n_{i}^{\max}\right)$ . In the oblique lattice γ_Obl_ = 105° and *n*_*i*_ varied between $n_{i}^{\min}$ and $n_{i}^{\max}$ where *i* ∈ {1, 2}. In addition, the distances between the centers of parallel tubules are given by the law of cosines $b_{1}=\sqrt{2a^{2}\left(1-\cos\gamma_{Obl}\right)}=1.59a$ or $b_{2}=\sqrt{2a^{2}\left[1-\cos\left(180-\gamma_{Obl}\right)\right]}=1.22a$ in the case of antiparallel tubules.

An alternative way of presenting the bundle of conical spiral tubules is by two lattices of parallel tubules

25 $${\mathrm{a⃗}}_{1}=2a\left(1,0\right)$$

26 $${\mathrm{a⃗}}_{2}=b_{j}\left(\cos\left(90+\frac{\gamma_{Obl}}{2}\right),\sin\left(90+\frac{\gamma_{Obl}}{2}\right)\right)$$

The two are shifted by (*a*, 0) from each other, and *j* ∈ {1, 2}. The tubules of one lattice are in antiparallel orientation to the tubules of the second lattice. To obtain antiparallel tubules, we apply a rotation around the *x*-axis by setting α = 180° in the Tait-Brian rotation.

Furthermore, to ensure the units align correctly within the lattice, a rotational correction around the *z*-axis (the γ angle in the Tait-Bryan convention) is applied based on the calculated center positions

27 $$\gamma =\arctan2\left({\mathrm{R⃗}}_{n}\cdot ŷ,{\mathrm{R⃗}}_{n}\cdot \mathrm{x̂}\right)\frac{180^{{}^{\circ}}}{\pi }$$

The illustrations of all the structures built using PDB ID3J6FandMO2Bwere created by ChimeraX software.^64^

## Supplementary Material



## Notes

The Supporting Information includes the derivation of the conical spiral tubule model, SVD analysis, additional SAXS curves, Cryo-TEM images, and tables listing all the best-fitted model parameters (PDF)
