# Supplementary material for: Modulating the Curvature of Protein Self-Assembled Spiral Nanotubules
Source: ACS Appl Mater Interfaces. 2025 May 12;17(20):29146–57. doi: 10.1021/acsami.5c01405 (PMC12100598; doi:10.1021/acsami.5c01405)
Supplement: Supplementary file 1 [file am5c01405_si_001.pdf]

Supporting Information
Modulating the Curvature of Protein Self-Assembled Spiral Nanotubules

Ariel Cohen,[†] Itai Ben-Nun,[†] Raviv Dharan,[†] Tamar Tayri-Wilk,[†] Asaf Shemesh,[†] Avi Ginsburg,[†] Abigail Millgram,[†] Yael Levi-Kalisman,[‡] Israel Ringel,[¶] and Uri Raviv[*,†,‡]

†*Institute of Chemistry, The Hebrew University of Jerusalem, Edmond J. Safra Campus, Givat Ram, 9190401, Jerusalem, Israel*

‡*The Harvey M. Krueger Family Center for Nanoscience and Nanotechnology, The Hebrew University of Jerusalem, Edmond J. Safra Campus, Givat Ram, Jerusalem 9190401, Israel*

¶*Institute for Drug Research, The School of Pharmacy, Faculty of Medicine, The Hebrew University of Jerusalem, Ein Karem, Jerusalem 9112102, Israel*

E-mail: uri.raviv@mail.huji.ac.il

Derivation of the Conical Spiral Tubule Model

The conical-spiral tubule model is derived from the parameterization of a conical helix. Initially, we define n as the number of helical turns, which may be non-integer, in the conical structure. The vertical pitch p describes the vertical separation between successive helical turns. The radii $R_{\max}$ and $R_{\min}$ correspond to the initial (larger) and final (smaller) spiral

radii, respectively. The radius decreasing rate per unit angle around the cone is:

$$C = \frac{R_{\max} - R_{\min}}{2\pi n} \tag{S1}$$

Using t as the parametric angular variable, the location parameters are expressed as follows:

$$x(t) = (R_{\max} - Ct)\cos t \implies \frac{\partial x}{\partial t} = (Ct - R_{\max})\sin t - C\cos t \tag{S2}$$

$$y(t) = (R_{\max} - Ct)\sin t \implies \frac{\partial y}{\partial t} = (R_{\max} - Ct)\cos t - C\sin t \tag{S3}$$

$$z(t) = \frac{p}{2\pi}t \implies \frac{\partial z}{\partial t} = \frac{p}{2\pi} = A \tag{S4}$$

To calculate the arc length S along the spiral from the start to a point defined by t_f, we perform a line integral:

$$\begin{aligned} S(t_f) &= \int_0^{t_f} \sqrt{\left(\frac{dr}{dt}\right)^2}\, dt \qquad \text{(S5)} \\ &= \int_0^{t_f} \sqrt{\left(\frac{\partial x}{\partial t}\right)^2 + \left(\frac{\partial y}{\partial t}\right)^2 + \left(\frac{\partial z}{\partial t}\right)^2}\, dt \\ &= \int_0^{t_f} \sqrt{\left((Ct - R_{\max})\sin t - C\cos t\right)^2 + \left((R_{\max} - Ct)\cos t - C\sin t\right)^2 + \left(\frac{p}{2\pi}\right)^2}\, dt \\ &= \int_0^{t_f} \sqrt{(Ct - R_{\max})^2 + C^2 + A^2}\, dt \\ &= \frac{1}{2C}\left[\left(C^2 + A^2\right)\operatorname{arcsinh}\left(\frac{C\left(Ct - R_{\max}\right)}{C\sqrt{C^2 + A^2}}\right) + (Ct - R_{\max})\sqrt{(Ct - R_{\max})^2 + C^2 + A^2}\right]_0^{t_f} \end{aligned}$$

For the conical spiral model, each i-th dimer's position and orientation are determined

as a function of t_i:

$$x_i = (R_{\max} - Ct_i)\cos t_i \tag{S6}$$

$$y_i = (R_{\max} - Ct_i)\sin t_i \tag{S7}$$

$$z_i = \frac{p}{2\pi} t_i \tag{S8}$$

$$A = \frac{p}{2\pi} \tag{S9}$$

$$S(t_i) = \frac{1}{2C}\left[\left(C^2 + A^2\right)\operatorname{arcsinh}\left(\frac{C(Ct - R_{\max})}{C\sqrt{C^2 + A^2}}\right) + (Ct - R_{\max})\sqrt{(Ct - R_{\max})^2 + C^2 + A^2}\right]_0^{t_i} \tag{S10}$$

The placement of each tubulin dimer, represented by t_i, is determined using a binary search within the range 0 to $2\pi n$. This search algorithm iteratively narrowed down the interval to locate the precise value of t_i that satisfies the equation $L_{\text{dim}} = S(t_i) - S(t_{i-1})$ within a convergence tolerance of $1 \times 10^{-14}\, nm$.

For the inner angles α, β, and γ, we find a cyclical dependency on t_i for β. The angle α, which defines the inclination from the z-axis to the x-y plane, is calculated as follows:

$$\Delta z_i = \frac{p}{2\pi} t_{i+1} - \frac{p}{2\pi} t_i = \frac{p}{2\pi}(t_{i+1} - t_i) = \frac{p}{2\pi}\Delta t_i \tag{S11}$$

$$\alpha_i = 90^\circ - \arcsin\frac{\Delta z_i}{L_{\text{dim}}} = 90^\circ - \arcsin\left(\frac{\frac{p}{2\pi}\Delta t_i}{L_{\text{dim}}}\right) = 90^\circ - \arcsin\left(\frac{p}{2\pi L_{\text{dim}}}\Delta t_i\right) \tag{S12}$$

$$\beta_i = \frac{180^\circ}{\pi} t_i \tag{S13}$$

$$\gamma_i = 0 \tag{S14}$$

where L_{dim} is the length of an individual dimer.

To summarize, the formulae for the conical spiral model are:

$$x_i = (R_{\max} - Ct_i)\cos t_i \tag{S15}$$

$$y_i = (R_{\max} - Ct_i)\sin t_i \tag{S16}$$

$$z_i = \frac{p}{2\pi}t_i \tag{S17}$$

$$C = \frac{R_{\max} - R_{\min}}{2\pi n} \tag{S18}$$

$$A = \frac{p}{2\pi} \tag{S19}$$

$$S(t_i) = \frac{1}{2C}\left[\left(C^2 + A^2\right)\operatorname{arcsinh}\left(\frac{C\left(Ct - R_{\max}\right)}{C\sqrt{C^2 + A^2}}\right) + \left(Ct - R_{\max}\right)\sqrt{\left(Ct - R_{\max}\right)^2 + C^2 + A^2}\right]_0^{t_i} \tag{S20}$$

$$\alpha_i = 90^\circ - \arcsin\left(\frac{p}{2\pi L_{\dim}}\Delta t_i\right) \tag{S21}$$

$$\beta_i = \frac{180^\circ}{\pi}t_i \tag{S22}$$

$$\gamma_i = 0 \tag{S23}$$

Supporting Figures and Tables

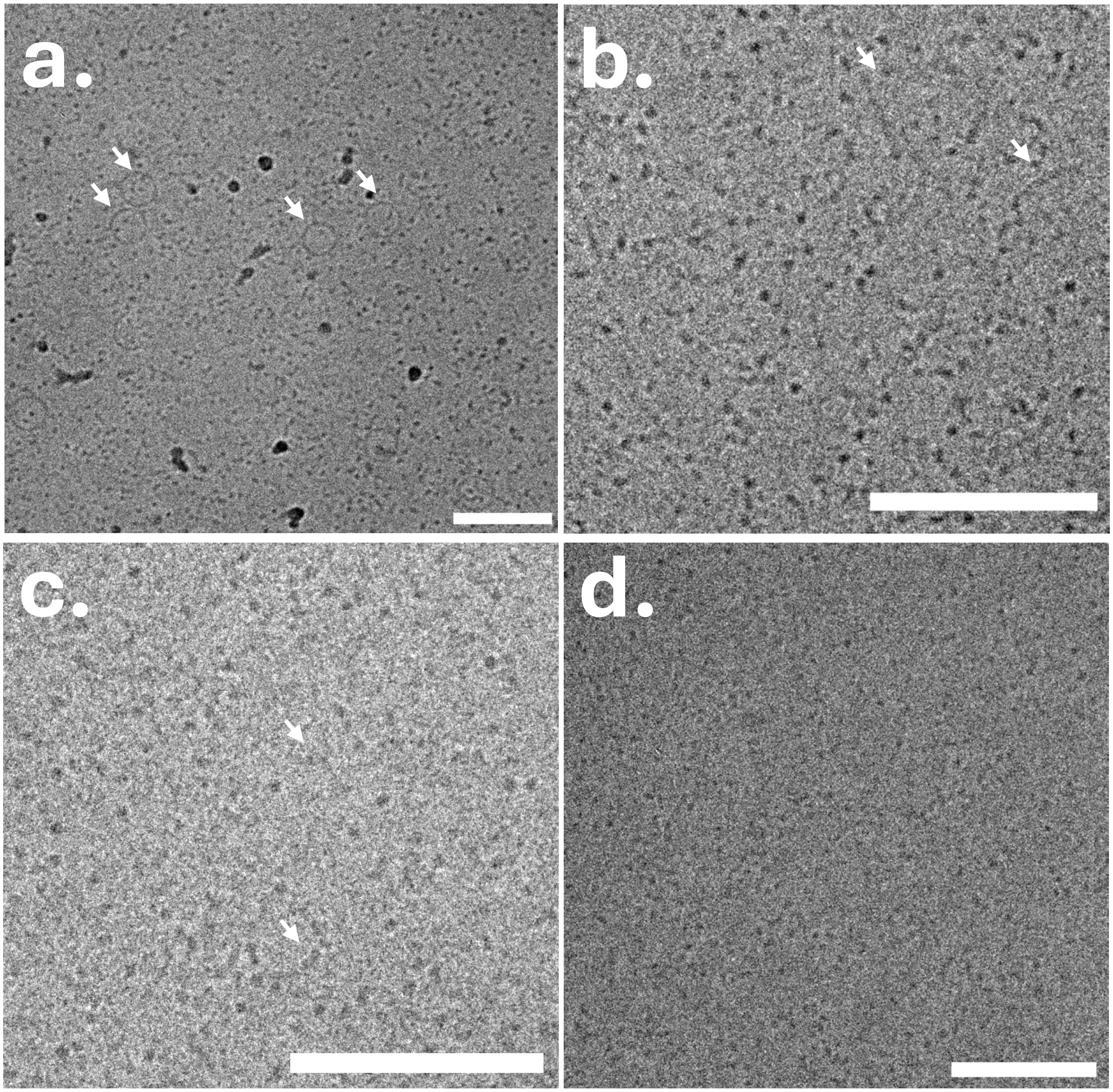

Figure S1: Selected Cryo-TEM images (in addition to those shown in Figure 1) of 100 μM GTP-tubulin incubated for 2 h at 9 °C without colchicine (a) with 1 μM colchicine (b), 2 μM colchicine (c) or 100 μM colchicine (d). scales bar equal 100 nm. Arrows show tubulin single rings in a or ring fragments in b. Three independent samples were measured in each case.

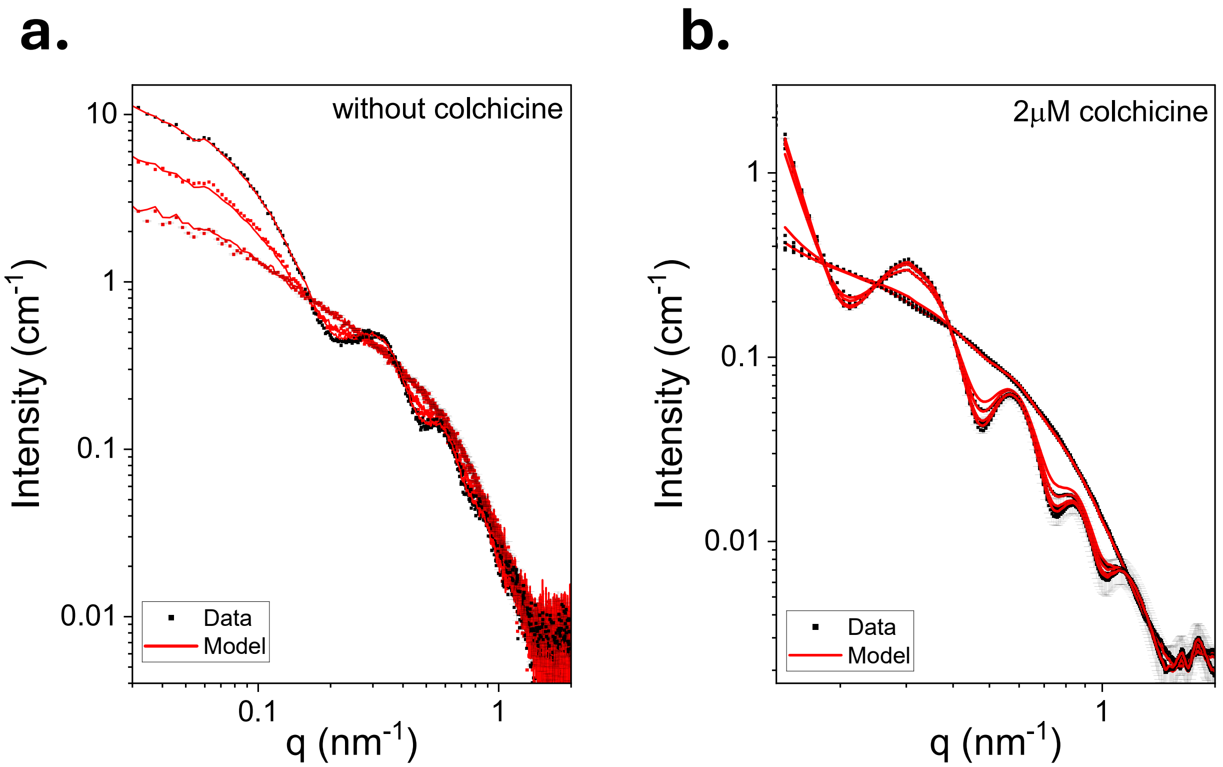

Figure S2: Supernatant-subtracted measured SAXS curves (black symbols) of 100 μM GTP-tubulin without colchicine (a.) and with 2 μM colchicine (b.), fitted to linear combinations (red curves) of the initial and final states of the kinetic reactions, taken from Figure 3. This Figure demonstrates the quality of the fitted two-state model to the data in Figure 3. Representative measurement black error bars are shown in transparent mode.

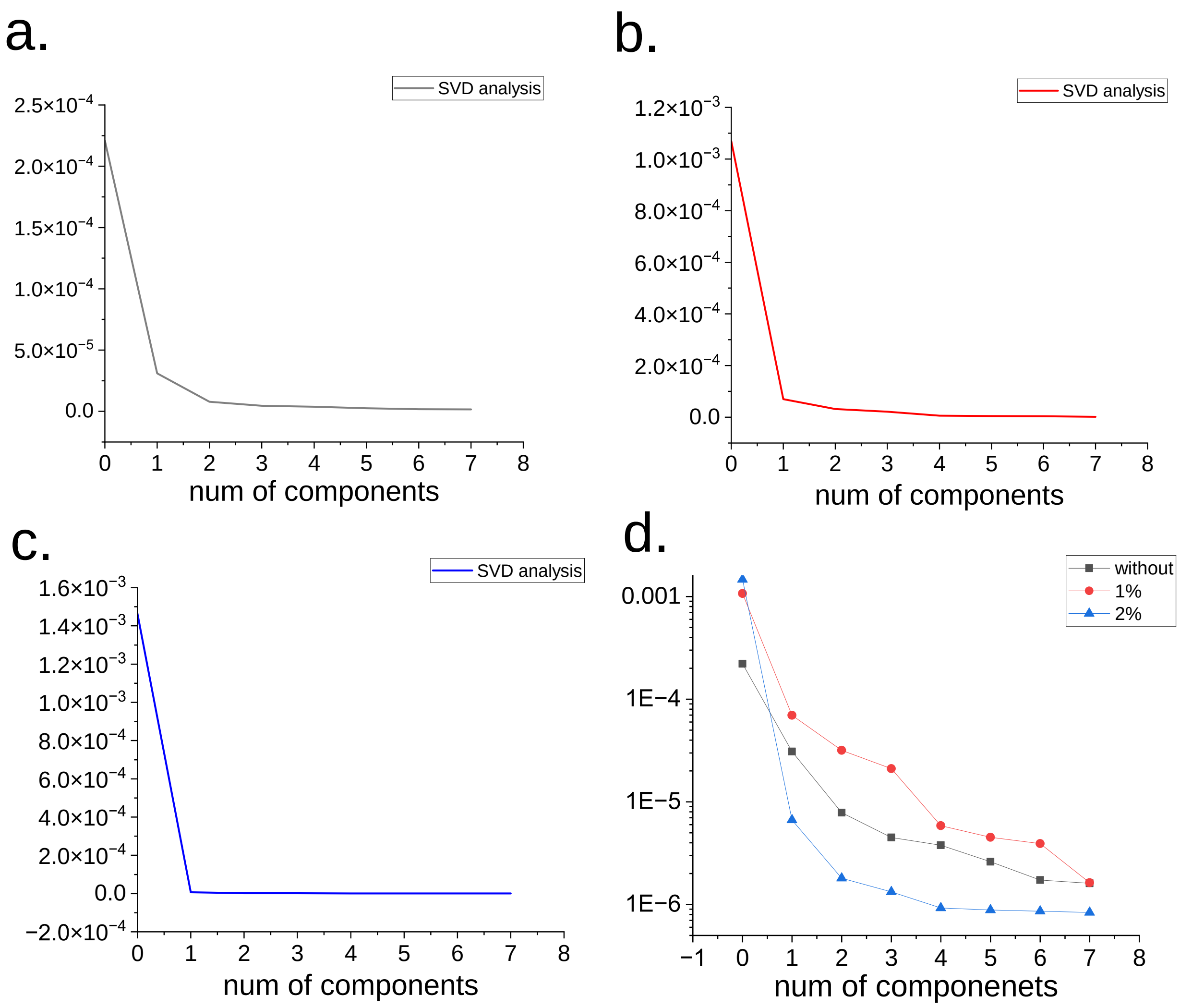

Figure S3: Singular value decomposition (SVD) analysis of the time-resolved data set from Figure 3 of 100 μM GTP-tubulin assembly in the absence of colchicine (a) and in the presence of 1 (b) or 2 μM colchicine (c). The SVD analyses are compared in a semilogarithmic plot in (d).

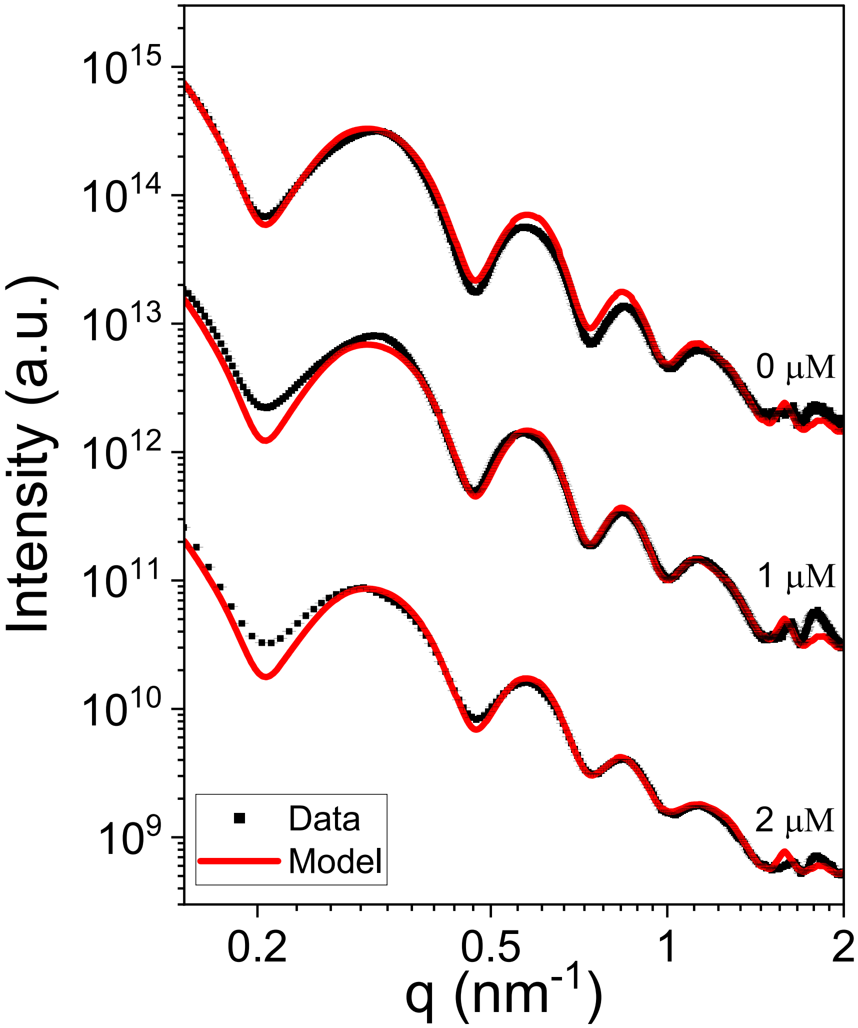

Figure S4: Supernatant-subtracted SAXS curves from 100 μM tubulin at 36 °C with the indication colchicine concentrations (black symbols and error bars in transparent mode), taken from Figure 2, fitted to a linear combination of free dimers and MT models with protofilament number distribution, describe in Table S1 (red curves).

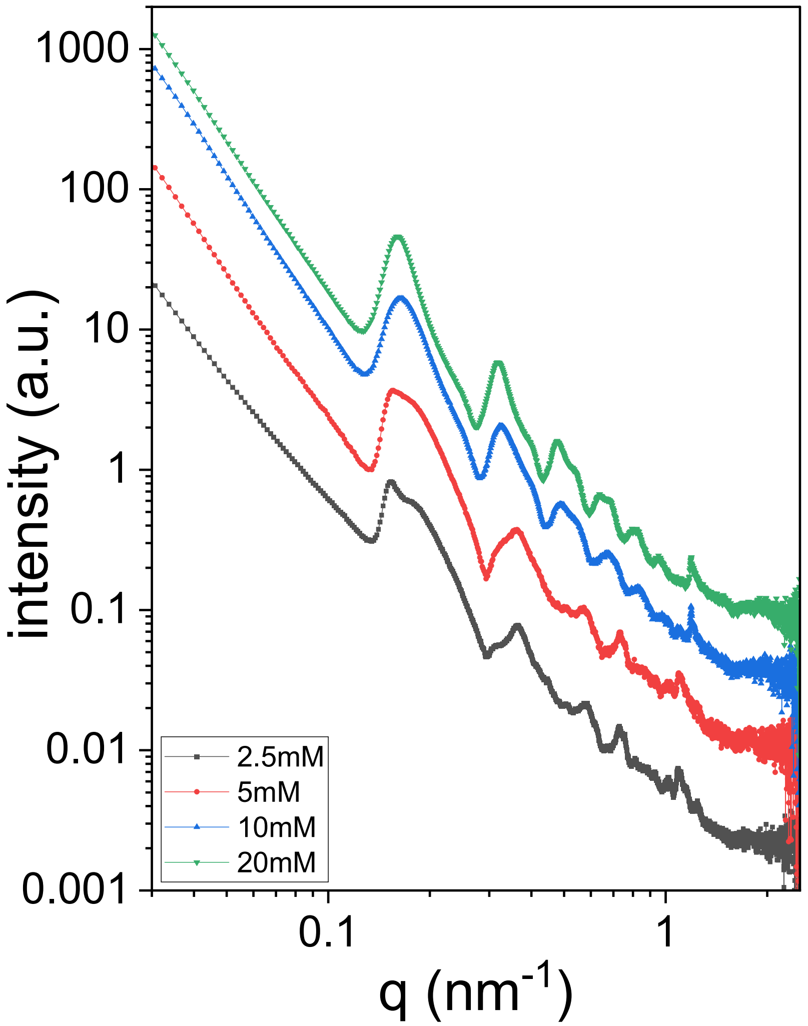

Figure S5: Supernatant-subtracted SAXS curves of 1:1 GDP-tubulin-colchicine molar ratio, mixed with different spermine concentrations at 36 °C. The colors of the error bars were matched to the color of the data. Three independent samples were measured in each case

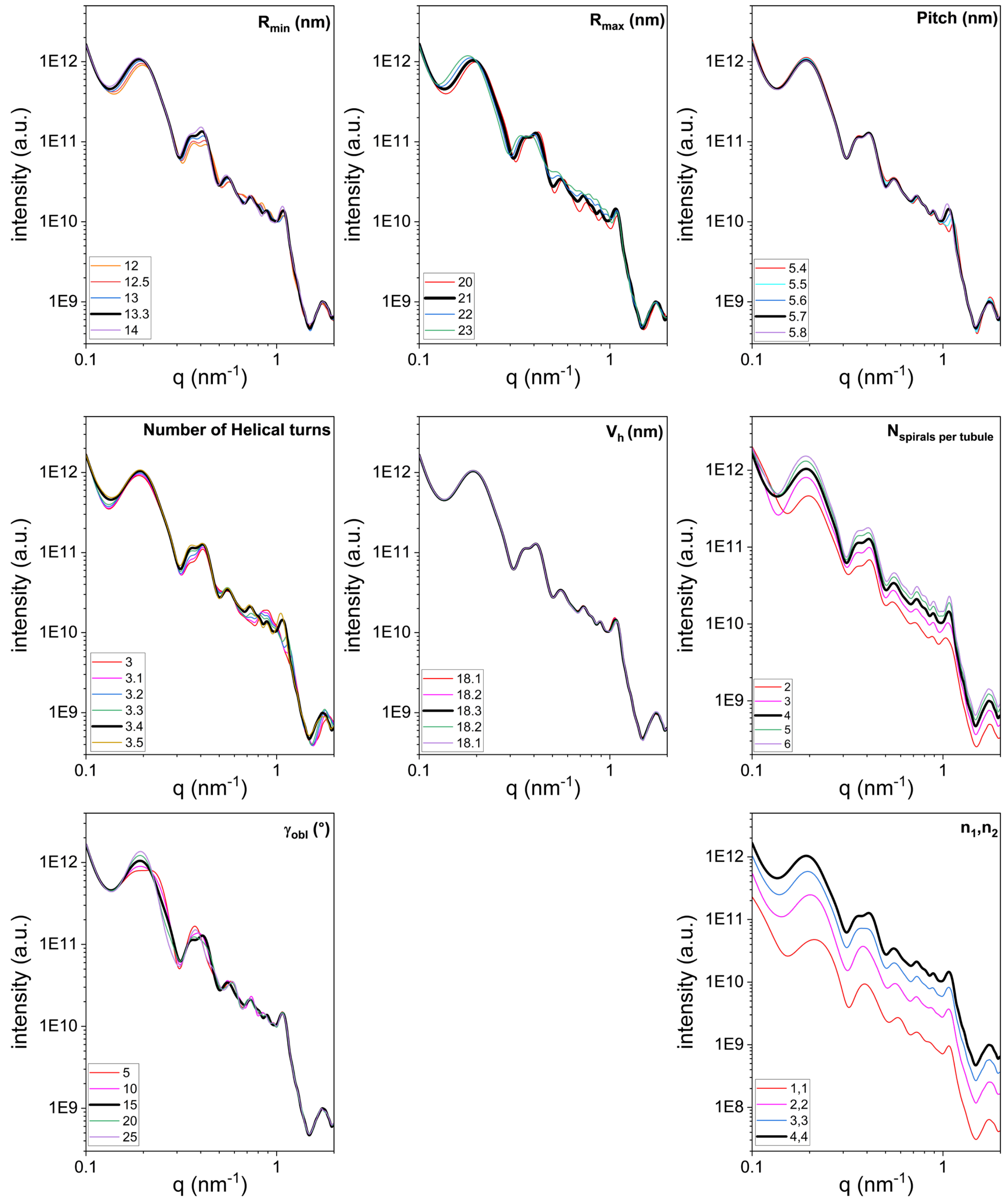

Figure S6: Effect of varying the indicated structural parameters, illustrated in Figure (CST cartoon), on the computed SAXS curve of conical spiral tubules, while fixing all the other parameters. The default parameters (bold black curves) are shown in Table S3.

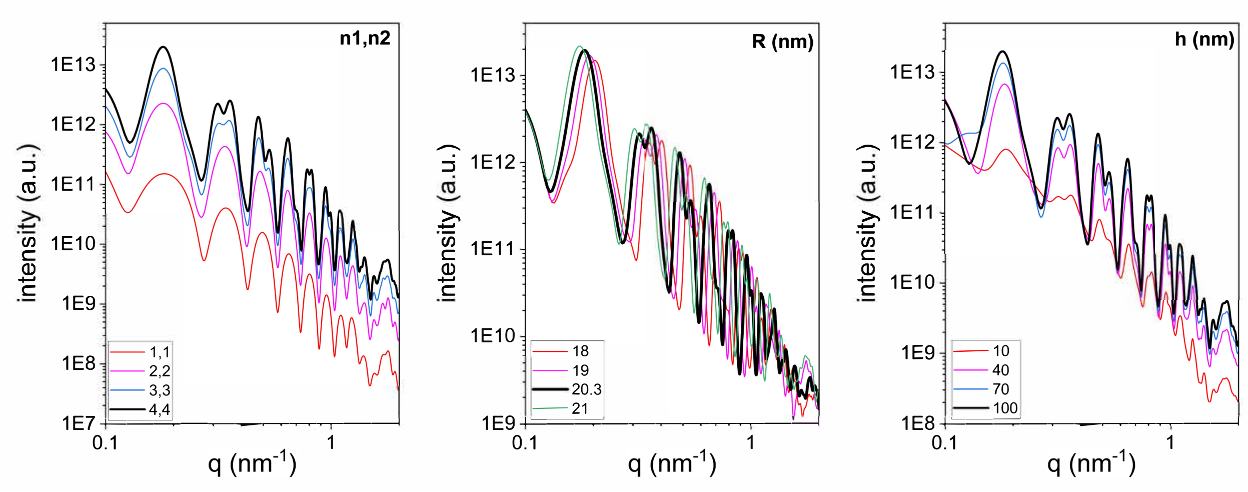

Figure S7: Effect of varying the indicated structural parameters of the inverted helical tubule model, while fixing all the other parameters. The default parameters (bold black curves) are shown in Table S5.

Table S1: MT protofilament number distribution as a function of colchicine concentration, following the analysis explained.[1]

Fraction				
Colchicine concentration (μM)	13 protofilaments	14 protofilaments	15 protofilaments	dimers
0	0.11±0.03	0.75±0.03	–	0.14±0.08
1	0.11±0.03	0.75±0.03	–	0.14±0.08
2	0.30±0.03	0.40±0.03	0.15±0.03	0.15±0.08

Table S2: The conical angle, θ, of the conical spirals, illustrated in Figure 8, the conical spiral width, $w = \frac{R_{\max} - R_{\min}}{sin\theta}$, and the conical spiral height, $h = \frac{R_{\max} - R_{\min}}{tan\theta}$, as a function of colchicine concentration.

Colchicine concentration (μM)	Conical Angle (degrees)	conical spiral width, w (nm)	conical spiral height, h (nm)
0	21.0 ± 0.9	19.5 ± 0.5	18.2 ± 0.5
20	21.6 ± 0.9	20.1 ± 0.5	18.2 ± 0.5
40	23 ± 1	19.7 ± 0.5	18.1 ± 0.5
80	24 ± 1	19.7 ± 0.5	18.0 ± 0.5

Table S3: The default parameters of the conical spiral tubules used in Figure S6.

$R_{\max}$ (nm)	$R_{\min}$ (nm)	Pitch (nm)	Number of Helical turns
21	13.3	5.7	3.4

V_h (nm)	n_1	n_2	γ_{obl} (degrees)	$N_{\text{Spirals per tubule}}$
18.3	4	4	105	4

Table S4: The structural parameters of the conical spiral tubule model.

Single Tubule Parameters						
Colchicine concentration (μM)	$R_{\max}$ (nm)	$R_{\min}$ (nm)	pitch (nm)	Number of helical turns	V_h (nm)	$N_{\text{Spirals per tubule}}$
0	21.5±0.2	14.5±0.1	5.60±0.05	3.4±0.1	18.2±0.1	4
20	21.5±0.2	14.3±0.1	5.60±0.05	3.4±0.1	18.2±0.1	4
40	21.0±0.2	13.3±0.2	5.70±0.05	3.4±0.1	18.0±0.1	4
80	21.0±0.2	13.0±0.1	5.70±0.05	3.4±0.1	18.0±0.1	6

Bundle Parameters				
Colchicine concentration (μM)	γ_{obl} (degrees)	n_1	n_2	a_1 (and a_2)
0	105	4	4	39.4
20	105	3	3	39.2
40	105	4	4	38.5
80	105	4	4	38.2

Table S5: Default inverted helical tubule parameters for Figure S7.

r (nm)	L_{dim} (nm)	Pitch (nm)	n_1	n_2	height (nm)	γ (degrees)
20.3±0.2	8.3±0.1	5.2±0.05	4	4	100	120

Table S6: The mass fraction of tubulin in main coexisting structures: free dimers, 5.2 nm height inverted helical tubule, and inverted helical tubule bundles.

Tubulin Mass Fraction			
Colchicine concentration (μM)	Inverted helical tubule bundles	Short single inverted helical tubules	Dimers
0	0.90±0.05	0.10±0.05	–
20	0.78±0.05	0.20±0.05	0.02±0.01
40	0.67±0.05	0.26±0.08	0.07±0.01
80	0.66±0.05	0.26±0.08	0.08±0.01

Table S7: Inverted helical tubule structural parameters.

Single tubule parameters			
Colchicine concentration (μM)	Radius (nm)	Height (nm)	Pitch (nm)
0	20.3±0.1	99	5.2±0.05
20	19.5±0.1	99	5.2±0.05
40	18.7±0.2	99	5.2±0.05
80	18.0±0.2	99	5.2±0.05

Hexagonal bundle parameters			
Colchicine concentration (μM)	$n_1 = n_2$	Center to center distance, a (nm)	γ (degrees)
0	4	49.8±0.2	120
20	4	48.2±0.2	120
40	4	46.6±0.2	120
80	4	45.2±0.2	120

References

(1) Ginsburg, A.; Shemesh, A.; Millgram, A.; Dharan, R.; Levi-Kalisman, Y.; Ringel, I.; Raviv, U. Structure of dynamic, Taxol-stabilized, and GMPPCP-stabilized microtubule. *J. Phys. Chem. B* **2017**, *121*, 8427–8436.
